# Major hepatectomy for primary hepatolithiasis: a comparative study of laparoscopic versus open treatment

**DOI:** 10.1007/s00464-018-6176-2

**Published:** 2018-04-03

**Authors:** Jian-xin Peng, Ling-zhi Wang, Jing-fang Diao, Zhi-jian Tan, Xiao-sheng Zhong, Zhi-peng Zhen, Gui-hao Chen, Jun-ming He

**Affiliations:** 1 Department of Hepatobiliary Surgery, Guangdong Province Traditional Chinese Medical Hospital, Guangzhou, 510120 People’s Republic of China; 2 Department of Anesthesia, The Second Affiliated Hospital, Guangzhou Medical University, Guangzhou, 510260 People’s Republic of China

**Keywords:** Laparoscopy, Major hepatectomy, Primary hepatolithiasis, Complication, Long-term outcomes

## Abstract

**Background:**

Due to higher technical requirements, laparoscopic major hepatectomy (LMH) for primary hepatolithiasis have been limited to a few institutions. This retrospective study was performed to evaluate the therapeutic safety, and perioperative and long-term outcomes of LMH versus open major hepatectomy (OMH) for hepatolithiasis.

**Methods:**

From January 2012 to December 2016, 61 patients with hepatolithiasis who underwent major hepatectomy were enrolled, including 29 LMH and 32 OMH. The perioperative outcomes and postoperative complications, as well as long-term outcomes, including the stone clearance and recurrence rate, were evaluated.

**Results:**

There was no difference of surgical procedures between the two groups. The mean operation time was (262 ± 83) min in the LMH group and (214 ± 66) min in the OMH group (*p* = 0.05). There is no difference of intra-operative bleeding (310 ± 233) ml versus (421 ± 359) ml (*p* = 0.05). In the LMH group, there were shorter time to postoperative oral intake ((1.1 ± 0.6) days versus (3.1 ± 1.8) days, *p* = 0.01) and shorter hospital stay [(7.2 ± 2.3) days versus (11.8 ± 5.5) days, *p* = 0.03] than the open group. The LMH group had comparable stone clearance rate with the OMH group during the initial surgery (82.8% vs. 84.4%, *p* = 0.86).

**Conclusions:**

LMH could be an effective and safe treatment for selected patients with hepatolithiasis, with an advantage over OMH in the field of less intra-operative blood loss, less intra-operative transfusion, less overall complications, and faster postoperative recovery.

Primary hepatolithiasis is defined as the presence of intra-hepatic calculi which is prevalent in Southeast Asia, but rare in Western countries [1]. Long-term hepatolithiasis may cause reduplicative cholangitis, secondary biliary stricture, liver cirrhosis, and even cholangiocarcinoma. The principles of primary hepatolithiasis treatment are to remove all biliary stones, to establish adequate drainage of the obstructed biliary system, and to resect atrophic liver parenchyma, which harbor bacteria and serve as a focus of infection [2]. Hepatectomy that can clean all the intra-hepatic ductal stones and remove strictured bile ducts within the resected liver segment(s), reducing the subsequent risks of recurrent stones and cholangiocarcinoma, seems to be the definitive and effective approach for this disease in selected patients [3].

With the development of laparoscopic technical approaches, laparoscopic hepatectomy (LH) has been utilized for the treatment of various liver diseases including benign and malignant liver tumors [4]. The laparoscopic approach follows the fundamental principles of open liver surgery. However, LH might be more difficult than open hepatectomy for hepatolithiasis due to the fact that patients with hepatolithiasis usually have aberrance of normal anatomical structures and perihepatic adhesions caused by chronic recurrent inflammation [5, 6].

Major hepatectomy was defined as the resection of 3 or more Couinaud’s liver segments, which is one of the most challenging and complex procedures encountered by the general surgeon, requiring considerable expertise [7]. There are a few papers reported major hepatectomy for hepatolithiasis treatment but no laparoscopic approach [8]. Laparoscopic major hepatectomy (LMH) have been limited to a few institutions because of the need for higher technical requirements. To date, there is no paper that reports the outcomes of comparing LMH with open major hepatectomy (OMH) for hepatolithiasis, and the feasibility and efficacy of LMH have not been fully evaluated. Therefore, this retrospective study was performed to evaluate the therapeutic safety, and perioperative and long-term outcomes of LMH versus OMH for hepatolithiasis.

## Materials and methods

From January 2012 to December 2016, 61 consecutive patients with intra-hepatic stones who underwent major hepatectomy at the Department of Hepatobiliary Surgery, Guangdong Province Traditional Chinese Medical Hospital were included. The indications for hepatic resection were as follows: bile duct stricture associated with stones, atrophy of the affected liver segments or lobes, presence of liver abscess, or the possible presence of cholangiocarcinoma. The clinical data for these patients were retrospectively analyzed. The parameters for comparison were perioperative outcomes and the rate of stone clearance. Postoperative follow-up was performed with CT and cholangiography. This study was conducted according to the Helsinki Declaration, and written informed consent was obtained.

### Surgical procedure

#### Procedure of laparoscopic major hepatectomies

Patients were placed in the reverse trendelenburg position under general anesthesia with endotracheal intubation. A CO_2_ pneumoperitoneum was established using an intra-abdominal pressure of 13–14 mmHg (1 mmHg = 0.133 kPa). According to the demands of the operation, the surgical bed was tilted to the right or left by 30°. A five-trocar method was used. The camera was placed 3 cm above the umbilicus. We first divided the round ligament using a harmonic scalpel and retracted it in a cranial direction. Subsequent medial or lateral retraction of the gallbladder resulted in access to the portal structures. The left and right hepatic arteries and their bifurcation were identified and isolated. The dissection began at the proper hepatic artery and extended cephalad, according to the demands of the operation, and the related arteries were divided and ligated. The distal hepatic artery was elevated and the portal vein was bluntly separated to its bifurcation. According to the demands of the operation, the left or right branch of the portal vein was ligated and divided. Obvious ischemic changes were usually seen after interruption of the segmental hepatic inflow. The round and falciform ligaments were divided to the level of the hepatocaval venous confluence and until the space between the right hepatic vein and the common trunk of the left and middle hepatic veins were separated. The line of resection followed the ischemic demarcation boundaries visible after the vasculature was divided. The position of the middle hepatic vein and the location of hepatolithiasis were confirmed by intra-operative laparoscopic ultrasound. The liver parenchyma was divided using harmonic scalpel, cavitron ultrasonic surgical aspirator (CUSA, Integra Lifesciences, USA), or Laparoscopic Peng’s multifunctional operative dissector (LPMOD, Shuyou Company, China), followed by the exposure of the stump of the left or right biliary duct. Intra-operative choledochoscope was applied to remove the bile duct stone as clean as possible. Stump of bile duct was sutured using 4-0 vicryl ine. A drainage tube was placed. The specimen was then placed in a specimen bag and removed through an incision below the umbilicus.

### Procedure of open major hepatectomies

The abdomen was explored through a subcostal incision with midline extension. The operative procedures of hepatectomy and intra-operative choledochoscope exploration were similar to those of laparoscopic group.

According to the demands of the operation, the left or right branch of the portal vein and hepatic artery were ligated and divided. Obvious ischemic changes were usually seen after interruption of the segmental hepatic inflow. The line of resection followed the ischemic demarcation boundaries visible after the vasculature was divided. The liver parenchyma was divided using harmonic scalpel, CUSA (Integra Lifesciences, USA), or Peng’s multifunctional operative dissector (LPMOD, Shuyou Company, China), followed by the exposure of the stump of the left or right biliary duct. Intra-operative choledochoscope was applied to remove the bile duct stone as clean as possible. Stump of bile duct was sutured using 4-0 vicryl line.

### Postoperative care and follow-up

All patients received the same postoperative monitoring and care, which included routine blood examinations and liver function tests.

The abdominal drainage was removed when the drainage fluid was serous and in the absence of bile leakage. For the patients with T-tubes, cholangiography was performed to make sure no stones were left before T-tubes were removed. If there were residual stones, they could be cleaned up through the T-tube sinus. All patients received a follow-up every 3–6 months at outpatient clinic. Routine physical examinations, liver laboratory tests, ultrasonography, and/or MRCP were performed during the follow-up.

### Statistical analysis

All statistical analyses were performed using dedicated software (SPSS version 17.0, SPSS Inc., Chicago, IL). Numeric variables were expressed as mean, standard deviation, or median (interquartile range). Categorical variables were compared by Chi-square or Fisher exact test, whereas the Wilcoxon rank sum test was used for continuous variables.

## Results

### Patients’ characteristics

From January 2012 to December 2016, 75 consecutive patients with intra-hepatic stones who underwent major hepatectomy were included. The patients who received major hepatectomy accompanying with hepaticojejunostomy (*n* = 14) were exclude. The other 61 patients met the inclusion criteria, including 29 LMH and 32 OMH. The preoperative data of these patients are summarized in Table 1. There were no significant differences of age, sex, biliary surgery history, extra-hepatic stones complicated, parenchymal atrophy, preoperative biliary drainage, and blood test results between the two groups.

**Table 1:** Preoperative data of the laparoscopic and open hepatectomy groups

	LH group (*n* = 29)	OH group (*n* = 32)	*p*
Age	61 (range 46–78)	59 (range 46–78)	0.104
Sex (male:female)	8:21	8:24	0.82
Previous biliary surgery	5 (17.2)%	9 (36%)	0.31
Complicated with extra-hepatic stones	24	26	0.88
Parenchymal atrophy	18	22	0.58
Liver cirrhosis	7	9	0.72
Portal hypertension	4	5	0.84
Chronic liver abscess	2	4	0.46
Preoperative biliary drainage	3	6	0.36
ERCP	0	1	0.34
PTCD	3	5	0.54
White blood cell (× 109/l)	6.8 ± 3.5	7.1 ± 3.7	0.84
Hemoglobin (g/l)	122 ± 12.1	125 ± 10.7	0.96
Platelet (× 109/l)	133 ± 58.3	141 ± 63.1	0.93
Alanine transaminase (U/l)	55.7 ± 40.4	61.3 ± 51.5	0.86
Total bilirubin (μmol/l)	37.2 ± 50.5	35.9 ± 48.1	0.97
Serum albumin (g/l)	38.1 ± 4.3	37.5 ± 5.2	0.82
Prothrombin time (s)	12.3 ± 2.2	12.7 ± 1.9	0.93

### Perioperative outcomes

There was no difference of surgical procedures between the two groups. 5 cases of right hemihepatectomy and 24 cases underwent intra-operative cholangioscopy in the LMH group, while 6 cases of right hemihepatectomy and 26 cases underwent intra-operative cholangioscopy in the OMH group. One patient (3.4%) was converted to open procedure in the LMH group because of severe abdominal adhesion. Perioperative outcomes are listed in Table 2. The mean operation time was (262 ± 83) min in the LMH group and (214 ± 66) min in the OMH group. There is no difference of intra-operative bleeding [(310 ± 233) ml vs. (421 ± 359) ml, *p* = 0.05]. Intra-operative transfusion was needed for 2 patients (6.9%) of the LMH group and 17 patients (21.9%) of the OMH group, but there is no statistical significance between them (*p* = 0.1). In the LMH group, there were shorter time to postoperative oral intake [(1.1 ± 0.6) days vs. (3.1 ± 1.8) days] and shorter hospital stay [(7.2 ± 2.3) days vs. (11.8 ± 5.5) days] than the OMH group (Table 2).

**Table 2:** Perioperative outcomes of the laparoscopic and open hepatectomy groups

	LH group (*n* = 29)	OH group (*n* = 32)	*p*
Operating time (min)	262 ± 83	214 ± 66	0.05
Intra-operative bleeding (ml)	310 ± 233	421 ± 359	0.05
Intra-operative transfusion (patient number)	2	7	0.1
Left hepatectomy	24	26	0.88
Right hepatectomy	5	6	0.88
Intra-operative cholangioscopy	27	29	0.72
T-tube insertion	26	29	0.9
Time to oral intake (day)	1.1. ± 0.6	3.1 ± 1.8	0.01
Postoperative hospital stay (day)	7.2 ± 2.3	11.8 ± 5.5	0.03

### Postoperative complications

There was no intra-operative mortality in the two groups. The morbidity of the LMH was significant lower than the OMH group (13.7% vs. 37.5%, *p* = 0.036) (Table 3). The LMH group had less wound infection (*p* = 0.026), subphrenic collection (*p* = 0.033) and pleural effusion (*p* = 0.018) rates than the OMH group. The patient who had subphrenic collection or pleural effusion recovered by percutaneous drainage. Although there was no statistical significance, the incidence of bile leak (*p* = 0.06), ARDS (*p* = 0.09), and intestinal obstruction (*p* = 0.09) were lower in the laparoscopy group. In both groups, one patient in each had portal vein thrombosis and both were successfully treated in conservative way. All the complications were improved at the time of discharge.

**Table 3:** The postoperative complications of the laparoscopic group and open groups

	LH group (*n* = 29)	OH group (*n* = 32)	*p*
Complication	4	12	0.036
Wound infection	0	5	0.026
Subphrenic collection/infection	1	7	0.033
Pleural effusion	2	8	0.017
Intra-abdominal fluid collection	1	4	0.198
Bile leak	1	6	0.06
Intraperitoneal bleeding	0	2	0.171
Portal thrombosis	1	1	0.94
ARDS	1	3	0.09
Intestinal obstruction	0	3	0.09

### Outcome of stone clearance and follow-up

The LMH group had comparable stone clearance rate with the OMH group during the initial surgery (82.8% vs. 84.4%, *p* = 0.86) (Table 4). Residual stones were removed by choledochoscopy via the T-tube 8 weeks after the first surgery.

**Table 4:** The stone clearance and recurrence rate

	LH group (*n* = 29)	OH group (*n* = 32)	*p*
Follow-up time (months)	27.2 ± 8.2	29.5 ± 10.7	0.93
Initial stone clearance rate (%)	24	27	0.86
Final stone clearance rate (%)	28	32	0.29
Recurrent stone	2	3	0.72

During a mean follow-up of 28 months (range 19–40 month), there were two patients (6.9%) who acquired recurrent stones in the LMH group and three (9.3%) in the OMH group. Four of these patients with stones in the CBD were treated by stone extraction with ERCP successfully. The other one with asymptomatic terminal hepatolithiasis in the OMH group was closely observed.

## Discussion

Primary hepatolithiasis often presents with pain, jaundice, recurrent pyogenic cholangitis, acute pancreatitis, and even cholangiocarcinoma that requiring active treatment after diagnosis. The aim of intra-hepatic stone treatment consists of complete removal of intra- and extra-hepatic stones, relief of obstruction, eradication of diseased tissues including atrophic liver parenchyma and biliary strictures, and construction of sufficient bile drainage. Hepatectomy is considered as the optimal treatment for this disease in selected patients for the efficiency of removal of the stones, the diseased bile ducts, and the damaged hepatic parenchyma lesions. In this way, hepatectomy could eliminate the risk of cholangiocarcinoma simultaneously.

With the development of laparoscopic techniques, LH has been widely applied for the treatment of various liver diseases including benign or malignant liver tumors. An increasing number of reports on LH with favorable results have been documented. Recently, some experienced surgeons have been trying to use LH for hepatolithiasis [9]. Laparoscopic left-sided hepatectomy for the treatment of hepatolithiasis had been reported with a favorable result of less intra-operative blood loss, intra-operative transfusion, overall complication, and faster postoperative recovery than open approach [10, 11]. But LMH for hepatolithiasis is not reported. As reported previously, major hepatectomy was defined as the resection of 3 or more Couinaud’s liver segments, while segmentectomy of 1 or 2 segments and non-anatomical wedge resection was classified as minor hepatectomy. In present studies, LMH includes left hemihepatectomy and right hemihepatectomy. We found that although it might cost more operation time, the LMH group have less intra-operative blood loss, transfusion, postoperative complications, and shorter hospital stay than open hepatectomy.

Bleeding control during the liver parenchyma dissection is the primary concern [12]. The Pringle maneuver was reported to control surgical blood loss in LMH. However, ischemic insult of the remnant liver is always a concern when this maneuver is used [13]. In our institution, we used the laparoscopic selective inflow and outflow occlusion methods to avoid bleeding. The hepatic artery, portal vein of affected side, and left vein or right vein were isolated and ligated individually before parenchyma dissection. The process can obviously decrease blood loss and ensure the blood supply of the remnant liver that is beneficial to gastrointestinal function recovery, so the LMH group has shorter oral intake time and postoperative hospital stay than the OMH. Laparoscopic right-sided hemihepatectomy for hepatolithiasis is more complicated than the left side for its repeated inflammation and perihepatic adhesions. It is only performed in a limited number of cases by experienced laparoscopic surgeons. In our study, due to the selective inflow and outflow occlusion methods, we achieved 5 cases of laparoscopic right-sided hemihepatectomy. One case was converted to laparotomy and none of them had complications.

Because of repeated infection of biliary tract and atrophy of liver parenchyma caused by intra-hepatic stones, there are many postoperative complications associated with hepatolithiasis. However, LH has less negative influence on the body, less invasive and fewer complications than laparotomy (13.7% vs. 37.5%, *p* = 0.036). Wound infection is the most common complication of hepatolithiasis after hepatectomy, but laparoscopic approach can reduce it sufficiently due to the small incisions. As reported previously, laparoscopic resection of liver also can reduced the inflammatory response compared with open resection, and we found that inflammation-related pleural effusion and ARDS are less in the LMH group [14]. Taken the advantage of magnification of the laparoscope, the smaller bile duct can be seen more clearly during operation, and the incidence of bile leakage is less in the LMH group, although the difference was not statistically valid.

Stone clearance rate is also an important indicator of liver resection for hepatolithiasis. Although it is still controversial that whether hepatectomy could be used to treat bilateral intra-hepatic stones, recent studies have suggested that resection of the dominantly affected side with postoperative cholangioscopic lithotomy might be a safe and effective approach for bilateral stones. We applied the intra-operative ultrasonography and cholangioscopy routinely. Although it is difficult to perform under laparoscopy, it can be well implemented by training. Intra-operative ultrasound can compensate for the lack of tactile defects in laparoscopy and can detect stones deep in the liver parenchyma [15]. Intra-operative choledochoscopy can be used for stone extraction and exploration and plays an important role in subsequent lithotripsy [16]. In our study, the stone clearance rate in LMH group was similar to that in LMH group.

In conclusion, our study indicates that LMH could be an effective and safe treatment for selected patients with hepatolithiasis, with an advantage over OMH in terms of less intra-operative blood loss, less intra-operative transfusion, less overall complications, and faster postoperative recovery. However, the reliability of the conclusions is limited because this study is only a single-institution retrospective research with limited cases. Additional well-designed, randomized controlled trials with a large sample size are needed to further confirm the role of LMH in the treatment of hepatolithiasis.
